# Calcined Xerogels of C/TiO_2_ Nanostructures for Solar-Driven Photocatalytic Hydrogen Production

**DOI:** 10.3390/gels11110911

**Published:** 2025-11-14

**Authors:** Yong Li, Hongpeng Zhang, Canni Zhuo, Xixi Sun, Jiaqi Gao, Yali Zhao

**Affiliations:** 1 Department of Materials Science and Engineering, Jinzhong University, Jinzhong 030619, China; 15635424073@163.com (H.Z.); zhuocanni111@163.com (C.Z.); k1224122chris@163.com (X.S.); gaojiaqi@jzxy.edu.cn (J.G.); 2 Shanxi Province New Multi-Functional Glass Technology Innovation Center, Jinzhong University, Jinzhong 030619, China

**Keywords:** carbon nanospheres, C/TiO_2_ nanostructures, photocatalysis, calcined xerogels, hydrogen production

## Abstract

The solar-driven water splitting for the production of renewable green hydrogen fundamentally relies on the exploration of efficient photocatalysts. Nanostructured TiO_2_ is widely recognized as a promising material for photocatalysis, yet it remains hindered by inadequate light harvesting and fast photogenerated carrier recombination. Herein, calcined C/TiO_2_ xerogels with yolk–shell and core–shell nanostructures (denoted as YS-C/TiO_2_ and CS-C/TiO_2_) were designed and fabricated via a typical sol–gel–calcination assisted approach. Thanks to the encapsulation of carbon nanospheres into TiO_2_, it effectively enhances light absorption, improves carrier separation, and lessens carrier recombination, making the well-designed YS-C/TiO_2_ composite display a remarkable hydrogen evolution rate of 975 µmol g^−1^ h^−1^ under simulated solar light irradiation and without the use of any co-catalyst, which is approximately 21.7 times that of the commercial TiO_2_. The work provides an efficacious design concept in developing nanostructured TiO_2_-based photocatalysts and in boosting broad photocatalytic applications.

## 1. Introduction

Energy shortage and environmental pollution are pressing scientific issues that need to be addressed urgently in contemporary society. Effectively harvesting and converting solar energy into thermal, electric, and chemical energy with more flexible applications is a promising approach to alleviate the current energy crisis; it is also an important way (e.g., CO_2_ photoreduction and degradation of VOCs driven by solar light) to achieve emission peak and carbon neutrality [1,2,3]. Photocatalysis technology, especially solar-driven water splitting for the production of renewable green hydrogen, is the current research hotspot in the field of energy transition [4,5].

Among numerous photocatalysts, titanium oxide (TiO_2_) stands out due to its favorable photo-stable, low-cost, and nontoxic properties, and is especially promising for the conversion of solar energy [6,7]. Nevertheless, intrinsic drawbacks in a broad bandgap of pristine TiO_2_ (3.2 eV) lead to poor solar energy utilization efficiency and fast recombination of photo-generated carriers [8,9]. To address these obstacles, multiple modification approaches have been developed, including morphology and structure regulation [10], cocatalyst deposition [11], heterojunction building [12], metal/nonmetallic-ion doping [13], and defect engineering [14] to enhance photocatalytic activity.

Carbon-based materials, as functional materials, have been widely used as adsorbents [15], catalysts [16], electrode materials [17], etc., thus becoming a research hotspot. In terms of photocatalysis, they can serve as photosensitizers to improve charge carrier transport efficiency and enhance light harvesting. At the same time, the superior electron mobility and efficient separation of photogenerated carriers confer exceptional electron-accepting and charge-transporting capabilities on carbon materials [18]. Furthermore, compared with other hybrid materials, carbon materials are stable, low-cost, and have the inherent advantage of being prepared from sustainable/eco-friendly raw materials (e.g., glucose, cellulose) [19]. In short, the combination of carbon materials and semiconductors to form hybrid nanostructures has attracted considerable attention recently.

Currently, carbon-based materials modified with TiO_2_ have attracted increasing interest from scientists [20,21]. The interaction between carbon and TiO_2_ is mainly reflected in two aspects: On one hand, carbon element doping regulates the bandgap of TiO_2_, which can greatly enhance the visible-light-driven photocatalytic performance of TiO_2_ [22]. Nonetheless, the preparation process of non-metal doping is relatively complex, restricting its practical application. On the other hand, carbon nanoparticles are loaded/deposited onto the outside of TiO_2_ or between particles to accomplish surface modification and interfacial sensitization [23]. For instance, graphitic carbon materials possess a large, conjugated structure. As visible-light photosensitizers, they endow TiO_2_ with excellent visible-light-driven photocatalytic activity through the charge transfer across the interfacial contact area [24]. Nevertheless, when carbonaceous materials coat the surface of TiO_2_, they will impede the capture of sunlight by TiO_2_, thus requiring specific control over the thickness of the carbon shell [25].

As is well-known, various morphological variations of TiO_2_ nanomaterials can be used to tune their shape-dependent physical properties [26,27]. Over the past few years, hollow nanostructured materials have drawn widespread interest within interdisciplinary research fields on account of their distinctive structural features and superior performances [28]. Because of their large specific surface area, clearly defined active sites, confined void volume, and adjustable mass-transfer velocity, hollow nanostructures can be used as superior catalysts in diverse catalytic processes [29]. Among them, for hollow TiO_2_ microspheres templated by carbon spheres, the hollow nanostructures are capable of elevating the light-absorption efficiency of TiO_2_ by means of multiple light scattering [30]. Moreover, more exposed TiO_2_ nanoparticles on the hollow nanostructures result in a larger specific surface area [31]. However, when fabricating TiO_2_ hollow nanostructures using carbon spheres as templates, the carbon spheres are inevitably burned off during the crystallization-calcination process of TiO_2_, and only a few studies have focused on attempting to retain the carbon core. The carbon core’s existence can not only enhance the stability and electrical conductivity of the C/TiO_2_ hollow structure but also promote efficient exciton transfer, with the expectation of achieving excellent photocatalytic activity.

Additionally, xerogel-derived composites have been widely applied in the field of photocatalysis. For example, bimetallic Co/Fe-MOX xerogels provided sufficient adsorption and active sites of Co for efficient photoreduction of CO_2_ [32]. Hybrid titania/silica xerogel dispersions were developed by incorporating silver nanoparticles to reinforce antimicrobial activity [33]. Cu_2-x_S/ZnO/carbon xerogel composites were facilitated an S-scheme heterojunction with carbon xerogel acting as a solid-state mediator, to enhance the photocatalytic degradation of organic pollutants [34,35]. Therefore, it is particularly important to investigate the structure–activity relationship between morphological structure and photocatalytic performance.

In this study, a novel strategy for constructing calcined C/TiO_2_ xerogel composites via employing a sol–gel–calcination assisted method, which was ingeniously designed to form a yolk–shell (YS-C/TiO_2_) and core–shell (CS-C/TiO_2_) nanostructures by cleverly encapsulating a core of carbon nanospheres with the TiO_2_ nano-shells. Within the YS-C/TiO_2_ and CS-C/TiO_2_ frameworks, the encapsulation of the carbon nanosphere may not only expeditiously transform the sunlight energy to thermal energy but also elevate the separation rate of the photo-induced carriers, thereby further boosting the photocatalytic H_2_ production performance. The present study offers a promising approach for constructing more stable and efficient nano-structured photocatalysts to boost large-scale photocatalytic applications.

## 2. Results and Discussion

### 2.1. Morphology Analysis of the Calcined C/TiO_2_ Xerogels

**Figure 1:**
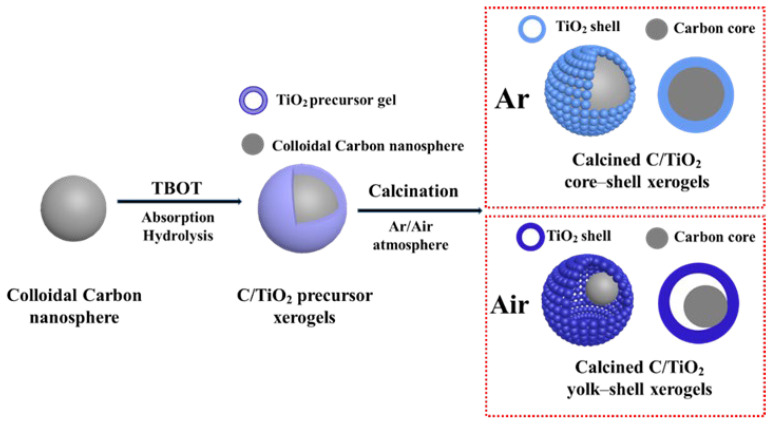
Schematic illustration of the synthesis procedure of calcined C/TiO_2_ xerogels.

The synthesis procedure for calcined xerogels of C/TiO_2_ yolk–shell and core–shell architectures include the following main steps, as shown in Figure 1: (1) hydrothermal preparation of monodisperse carbon sphere colloids (MCSs) (as the starting template), (2) the formation of C/TiO_2_ precursor xerogels with absorption and hydrolysis processes of TBOT onto the MCSs, and (3) calcination process to obtain a series of nanostructures (controlling calcination atmosphere and time).

**Figure 2:**
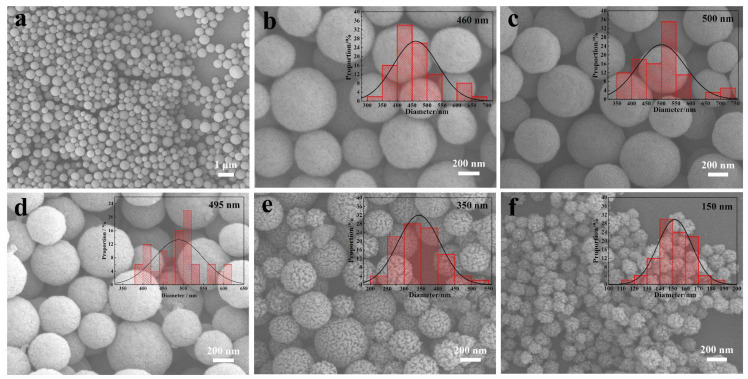
Morphology analysis of the calcined C/TiO_2_ xerogels. SEM images and the corresponding particle size distribution maps of (**a**,**b**) MSCs, (**c**) C/TiO_2_ precursor, (**d**) CS-C/TiO_2_, (**e**) YS-C/TiO_2_, and (**f**) H-TiO_2_.

The above steps were closely monitored by SEM and TEM measurements, capturing detailed structural and morphological changes during the preparation process. Figure 2 exhibits the corresponding SEM images and particle-size distribution maps of the synthesized structures from each step.

Carbon spheres, as classic hard templates, have found widespread use in designing and synthesizing hollow core–shell and other micro/nanostructures [36]. In a 180 °C hydrothermal environment, glucose forms carbon sphere colloids through dehydration, polycondensation, and carbonization processes [37]. However, severe agglomeration occurs between the particles, which impairs the subsequent uniform coating of TiO_2_ precursors on their surface. To solve this problem, well-monodispersed and homogeneous colloidal carbon spheres were effectively prepared by adding a small amount of sodium polyacrylate (PAANa; 0.2 mg mL^−1^) under the same hydrothermal carbonization (HTC) conditions [38], as shown in Figure 2a,b. Numerous pores are scattered across the surfaces of the carbon nanospheres. Beyond increasing the specific surface area of the carbon spheres and optimizing the structure of their pores, this structure further facilitates the mass-transfer process and improves the carbon nanospheres’ ability to adsorb precursors (C/TiO_2_ precursor).

Because the carbon template is not burning in an Ar atmosphere, it leads to a rigid TiO_2_ shell, and only a small volume change in C/TiO_2_ core–shell (CS-C/TiO_2_) nanostructure (Figure 2d; 495 nm) with respect to C/TiO_2_ precursor (Figure 2c; 500 nm); meanwhile, the surface becomes rougher owing to the crystallization of TiO_2_. However, when calcined in an air atmosphere, the carbon template begins to burn and the TiO_2_ shell crystallizes.

The difference between the TiO_2_ shell formation rate and carbon nanosphere template removal rate results in the formation of internal voids (Figure 2e,f). Owing to the reduction in inner MCSs and the shrinkage of outer TiO_2_, the volume of the inner cavity changes, and the surface becomes coarser, similar to a walnut shell. This indirectly proves the development of yolk–shell nanostructure (YS-C/TiO_2_). As the calcination time increases, the inner MCSs become smaller, and the outer TiO_2_ shell shrinks severely, leading to a drastic change in surface morphology and particle size, forming the hollow (H-TiO_2_) nanostructure (Figure 2f). Consequently, the mean diameter of C/TiO_2_ precursor xerogels reduces notably, starting from 500 nm (Figure 2c; C/TiO_2_ precursor) to 350 nm (Figure 2e; YS-C/TiO_2_), further to 150 nm (Figure 2f; H-TiO_2_).

**Figure 3:**
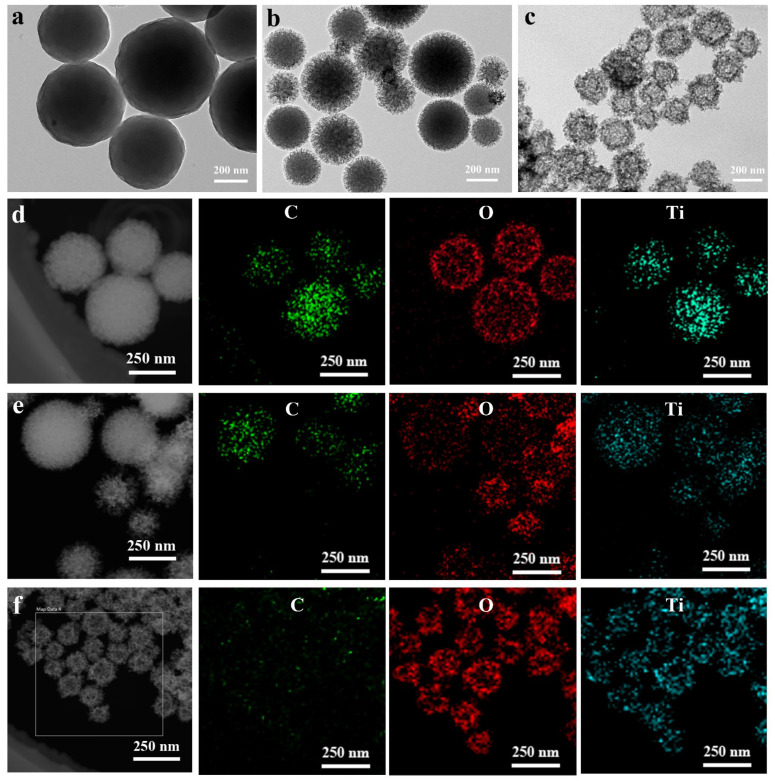
Morphology and elemental distribution analysis of the calcined C/TiO_2_ xerogels. TEM images of (**a**) CS-C/TiO_2_, (**b**) YS-C/TiO_2_, (**c**) H-TiO_2,_ and EDX elemental mappings of (**d**) CS-C/TiO_2_, (**e**) YS-C/TiO_2_, and (**f**) H-TiO_2_.

More importantly, TEM and EDX elemental mappings can intuitively distinguish the change process of the structure and morphology of the calcined C/TiO_2_ xerogels. As depicted in Figure 3a, the C/TiO_2_ precursor was subjected to calcination in an Ar atmosphere, which ultimately resulted in the formation of C/TiO_2_ core–shell structure (CS-C/TiO_2_) composite. However, when calcined in air, certain voids are generated inside the composite due to the combustion and decomposition of the carbon core, thus forming a composite with a yolk–shell structure (YS-C/TiO_2_), as illustrated in Figure 3b. The coexistence of carbon core and voids in YS-C/TiO_2_ hybrid may be beneficial to sunlight harvesting, and better support the multiple light-scattering process of the TiO_2_ shells. Continuous calcination in air until the MCS’s core achieves complete breakdown eventually results in the development of a hollow structure (H-TiO_2_), as exhibited in Figure 3c. Moreover, elemental mapping images (Figure 3d–f) distinctly demonstrate the CS-C/TiO_2_, YS-C/TiO_2_, and H-TiO_2_ nanostructures, with C predominantly found in the core while O and Ti are mainly located on the outside.

### 2.2. Structural and Light Absorption Properties of the Calcined C/TiO_2_ Xerogels

**Figure 4:**
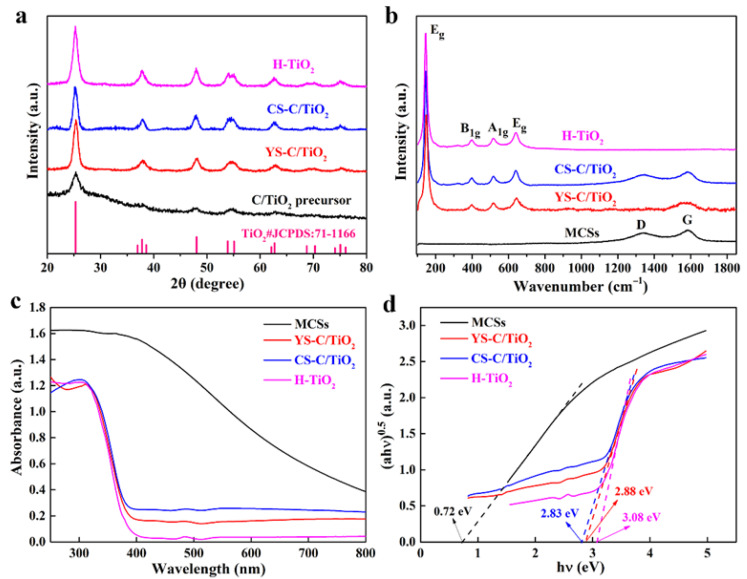
Structural and light absorption properties. (**a**) XRD patterns, (**b**) Raman spectra, (**c**) UV–Vis DRS spectra, and (**d**) Tauc curves of various calcined C/TiO_2_ xerogels.

The phase and crystal structures of calcined C/TiO_2_ xerogels were examined by XRD, as depicted in Figure 4a. The C/TiO_2_ composites exhibit characteristic peaks at 25.3°, 36.9°, 37.9°, 38.6°, 48.0°, 53.9°, 55.1° and 62.1°, assignable to the anatase phase of TiO_2_ (JCPDS#71-1166) after calcination at 500 °C in air (YS-C/TiO_2_ and H-TiO_2_) or Ar atmosphere (CS-C/TiO_2_). Apparently, there are no characteristic peaks associated with carbon species of calcined xerogels, that is because carbon species are generally synthesized from hydrocarbons, exhibiting almost amorphous characteristics. Notably, as the calcination time is prolonged, the intensity of the diffraction peaks of H-TiO_2_ enhances correspondingly, implying an improvement in the crystallinity of TiO_2_. Furthermore, Raman spectroscopy confirmed the coexistence of carbon and TiO_2_ in the C/TiO_2_ composites. In Figure 4b, for YS-C/TiO_2_ and CS-C/TiO_2_ samples, apart from the four anatase characteristic peaks of TiO_2_, two additional Raman peaks at 1348 cm^−1^ and 1582 cm^−1^ are associated with the disorder (D) and graphite (G) bands of carbon species [39], signifying that TiO_2_ was successfully loaded onto carbon core, and the interfacial interaction may accelerate the separation and migration of the photo-induced carriers [40]. In addition, the absence of D or G bands in H-TiO_2_ indicates that the carbon core has been completely burned off. TGA was carried out to further confirm the carbon content of various calcined C/TiO_2_ xerogels (Figure S1). Between 200 and 500 °C, significant weight loss occurred for the CS-C/TiO_2_ and YS-C/TiO_2_ samples, which corresponded to the disintegration of the carbon core, and demonstrated the total weight loss of 30.1% for CS-C/TiO_2_ and 6.4% for YS-C/TiO_2_ until 600 °C. In the case of H-TiO_2_, it revealed minor weight loss from 200 to 600 °C, implying that the carbon core has completely decomposed and formed hollow TiO_2_.

The UV–Vis DRS was conducted to investigate the light-absorption features for calcined C/TiO_2_ xerogels. As depicted in Figure 4c, the bare MCSs show a broad absorption spanning the UV and visible regions, while C/TiO_2_ hybrids display a comparable absorption edge close to 380 nm, presenting the typical bandgap of the anatase phase TiO_2_ [41]. Both CS-C/TiO_2_ and YS-C/TiO_2_ samples exhibit strengthened absorption within the VIS region in comparison with H-TiO_2_, which is ascribed to the introduction of the MCSs core. Consequently, from the Tauc curves given in Figure 4d, the optical bandgap of H-TiO_2_ stands at 3.08 eV, whereas that of YS-C/TiO_2_ and CS-C/TiO_2_ is slightly reduced to 2.88 and 2.83 eV, respectively. Therefore, the encapsulation of carbon into TiO_2_ enhances its photocatalytic activity by improving visible light harvesting.

### 2.3. Charge Separation and Transfer over the Calcined C/TiO_2_ Xerogels

**Figure 5:**
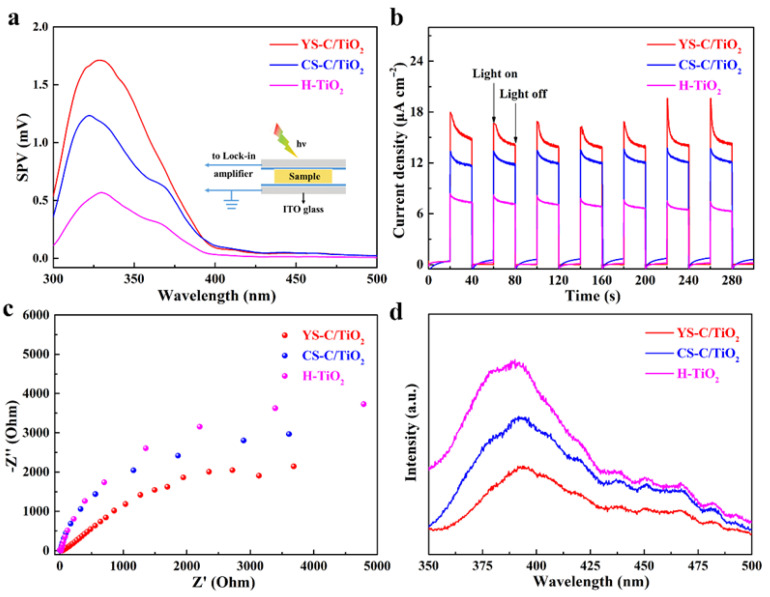
Charge separation/transfer on photocatalysts. (**a**) Surface photovoltage (SPV) spectra, (**b**) Transient photocurrent response curves, (**c**) EIS plots, and (**d**) PL spectra of various calcined C/TiO_2_ xerogels.

The fast separation and transport of photo-generated carriers serves as a critical factor influencing the photocatalytic activity of calcined C/TiO_2_ xerogels; thus, surface photovoltage (SPV) spectroscopy, photoelectrochemical test and electrochemical impedance spectroscopy (EIS) measurement, and photoluminescence (PL) spectroscopy were utilized to characterize the separation and transport performance of the photo-induced carriers within YS-C/TiO_2_, CS-C/TiO_2_, and H-TiO_2_ photocatalysts (Figure 5). Concretely, the SPV response primarily results from the photo-generated charge separation via diffusion on photocatalysts [42]. Figure 5a demonstrates that all samples possess clearly positive SPV peaks, ascribed to band-to-band transitions, which implies typical features of an n-type semiconductor [43]. The C/TiO_2_ composite exhibits a higher SPV intensity than H-TiO_2_, indicating an intense interaction between the MCS’s core and TiO_2_ shell during the calcination process, which exerts a primary function in the carrier transfer step. Compared with H-TiO_2_, YS-C/TiO_2_, and CS-C/TiO_2_ exhibit a notable photocurrent response (Figure 5b), which reveals that the improved separation of electron–hole pairs is attributed to the introduction of carbon core in the hybrids. The YS-C/TiO_2_ presents the maximum photocurrent intensity, which signifies the excellent photo-induced carrier separation efficiency among the C/TiO_2_ nanostructures. Similarly, the YS-C/TiO_2_ displays a smaller arc in the EIS spectra relative to the other catalysts (Figure 5c), signifying a reduced carrier transfer resistance.

Moreover, the PL spectra show that C/TiO_2_ composites exhibit a significant PL quenching effect, which is partly due to the recombination of photo-generated carriers. The PL intensity of YS-C/TiO_2_ exhibits notably lower values in comparison with that of H-TiO_2_ and CS-C/TiO_2_ (Figure 5d), demonstrating the most pronounced quenching. This reduced recombination of photo-generated carriers may enhance the photocatalytic performance of YS-C/TiO_2_, given that more charge carriers can be utilized in photocatalytic H_2_ production. The above discussions suggest that the encapsulation of carbon core into TiO_2_ enhances light absorption, improves charge transfer, and reduces charge recombination, making calcined C/TiO_2_ xerogels a promising composite for efficient photocatalytic hydrogen evolution.

### 2.4. Photocatalytic H_2_ Evolution Performance and Mechanism for Calcined C/TiO_2_ Xerogels

**Figure 6:**
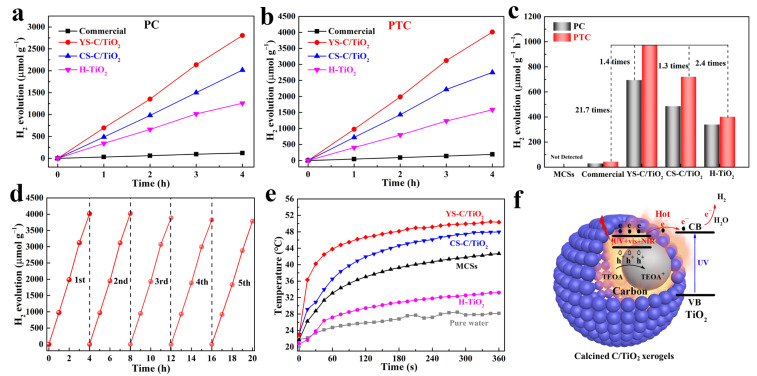
Photocatalytic H_2_ evolution performance and mechanism for calcined C/TiO_2_ xerogels. (**a**,**b**) H_2_ evolution amounts of different catalysts at different irradiation times with (PC) and without (PTC) cooling the reaction solutions; (**c**) Comparison of the H_2_ evolution rates of different catalysts between PC and PTC; (**d**) The photostability testing of YS-C/TiO_2_ over five consecutive cycles; (**e**) Time-dependent temperature curves of different catalysts suspension (2 mg mL^−1^) under simulated sunlight irradiation; and (**f**) The mechanism of photocatalytic H_2_ evolution for YS-C/TiO_2_.

Photocatalytic H_2_ evolution performances of commercial TiO_2_ and calcined C/TiO_2_ xerogels under simulated solar-light illumination (AM 1.5G) were investigated by employing TEOA as the sacrificial agent and without co-catalyst Pt. Blank tests revealed that no H_2_ could be detected without light irradiation or a catalyst, nor when pure MCSs were irradiated under simulated solar light. As shown in Figure 6a, due to severe photogenerated charge recombination and low surface-reaction kinetics, commercial TiO_2_ exhibits a relatively low hydrogen evolution rate of only 31 µmol g^−1^ h^−1^. After optimizing the calcination process, the C/TiO_2_ composites with retained (or partially retained) carbon cores demonstrate excellent hydrogen evolution activity. In particular, YS-C/TiO_2_ exhibits the highest photocatalytic (PC) hydrogen evolution activity, with a hydrogen evolution rate of up to 696 μmol g^−1^ h^−1^. After removing the circulating-water cooling system, its hydrogen evolution performance (PTC) is significantly enhanced (Figure 6b). Among the samples, YS-C/TiO_2_ also achieves the highest hydrogen evolution rate (975 μmol g^−1^ h^−1^), which is approximately 21.7 times that of commercial TiO_2_ (45 μmol g^−1^ h^−1^) (Figure 6c).

To highlight the advantages of the YS-C/TiO_2_ catalyst, a comparison of its photocatalytic hydrogen evolution activity with that reported for other similar systems is summarized in Table S1. YS-C/TiO_2_ demonstrates excellent hydrogen evolution efficiency under simulated solar light irradiation, even in pure water splitting (102 μmol g^−1^ h^−1^, without a sacrificial agent), and outperforms some reported TiO_2_-based photocatalysts. This fully underscores the importance of the MCS’s core: the heat resulting from the photothermal effect of the MCSs is sustainably transferred from the inner MCSs to the TiO_2_ shell, promoting the activation of surface reaction sites. Furthermore, YS-TiO_2_ also exhibits outstanding hydrogen evolution stability (Figure 6d). No significant decrease in its hydrogen evolution activity is observed during the 20 h cyclic test. After five cycles, the hydrogen evolution activity only decreases by 5.6%, which can be attributed to trace losses during the cyclic experiments. Further SEM and XRD analyses (Figure S2) confirmed that both the morphology and crystal phase remained unchanged after stability tests, indicating good stability and recyclability of the catalyst. The excellent photocatalytic H_2_ production performance over YS-C/TiO_2_ stems from the photothermal effect of the carbon core in the yolk–shell nanostructure. As displayed in Figure 6e, under simulated solar-light illumination, the temperature of the YS-C/TiO_2_ suspension increases immediately (within 1 min) and rises to 49.9 °C after 5 min. This rapid temperature response is ascribed to the photothermal effect of the MCSs, which is capable of expeditiously transforming sunlight into thermal energy. Furthermore, due to the low thermal conductivity of the TiO_2_ shell, the MCSs core is encapsulated inside the TiO_2_ shell, which significantly restrains heat loss to the surrounding environment and fully demonstrates the advantage of the yolk–shell nanostructure in heat collection.

Therefore, a possible mechanism of photocatalytic hydrogen evolution for calcined C/TiO_2_ xerogels is proposed, as shown in Figure 6f. Encapsulating MCSs in a TiO_2_ shell to build a yolk–shell nanostructure with high light-scattering capability could serve as an appropriate approach for enhancing photocatalytic activity. First, the bandgaps of H-TiO_2_ and MCSs are 3.08 eV and 0.72 eV, respectively, demonstrating that H-TiO_2_ is only capable of responding to UV light, whereas MCSs are responsive to the full spectrum. When simulated solar light irradiates the YS-C/TiO_2_, the carbon core can simultaneously absorb incident light and scattered light in the near-field of the TiO_2_ shell surface, generating charge carriers. With the heat generated by the photothermal effect of the carbon core, high-energy hot electrons are transferred to the conduction band (CB) of the TiO_2_ shell to drive the surface hydrogen evolution reaction, while holes positioned at the HOMO (highest occupied molecular orbital) energy level of the carbon core are captured by triethanolamine (TEOA). Second, the heat accumulation resulting from the photo-thermal excitation in the carbon core is able to further speed up the surface hydrogen evolution reaction. Finally, the yolk–shell nanostructure between inner MCSs and TiO_2_ shells forms a C/TiO_2_ heterojunction that offers more free pathways for the separation and transfer of photo-generated carriers, and fundamentally improves the photocatalytic activity for water-splitting of hydrogen evolution over YS-TiO_2_. The main photocatalytic process is described as follows:

(1) $$C/TiO_{2}+hv\overset{Sunlight}{\to }C\left(e_{CB}^{-},h_{VB}^{+}\right)/TiO_{2}$$

(2) $$C\left(e_{CB}^{-},h_{VB}^{+}\right)/TiO_{2}\overset{Chargetransfer}{\to }C\left(e_{CB}^{-},h_{VB}^{+}\right)/TiO_{2}\left(e_{CB}^{-}\right)$$

(3) $$TiO_{2}\left(e_{CB}^{-}\right)+{2H}_{2}O\to TiO_{2}+H_{2}+{2OH}^{-}$$

(4) $$\left(h_{VB}^{+}\right)+{OH}^{-}\to C+•OH$$

(5) $$C\left(h_{VB}^{+}\right)/•OH+TEOA\to C+{TEOA}^{+}$$

## 3. Conclusions

In summary, we successfully designed and synthesized calcined xerogels of C/TiO_2_ with yolk–shell and core–shell nanostructures using a sol–gel–calcination assisted method as promising photocatalysts for hydrogen evolution. Taking advantage of the photothermal effect of inner carbon nanospheres and the structural merits of yolk–shell and core–shell, the utilization of sunlight by the composites was promoted, and the C/TiO_2_ heterojunction was also beneficial for carrier transport and heat transfer. Among the fabricated photocatalysts, YS-C/TiO_2_ exhibits the maximum photocatalytic hydrogen evolution performance, up to 975 µmol g^−1^ h^−1^ under simulated solar-light illumination (AM 1.5G) without using a co-catalyst, which is approximately 21.7 times that of the commercial TiO_2_. The design strategy demonstrated here provides valuable insights into nanostructure regulation for efficient solar-driven photocatalytic hydrogen production, contributing to the development of sustainable energy and environmental applications.

## 4. Materials and Methods

### 4.1. Materials

Glucose (C_6_H_12_O_6_, Sinopharm Chemical Reagent (Shanghai, China), AR), tetrabutyl titanate (TBOT, Aladdin (Shanghai, China), 98%), absolute ethanol (C_2_H_5_OH, Sinopharm Chemical Reagent, AR), sodium polyacrylate (PAANa, Aladdin, AR), and triethanolamine (TEOA, Aladdin, AR) were employed without any purification.

### 4.2. Synthesis of Monodisperse Carbon Sphere Colloids

Monodisperse carbon sphere colloids (MCSs) were synthesized by a glucose hydrothermal method assisted with sodium polyacrylate (PAANa) [38]. Typically, 12 g of glucose was dissolved in 50 mL of deionized water. Next, 15 mg PAANa was added to the solution. Subsequently, the solution was transferred into a 100 mL autoclave and held at 180 °C for 8 h. The brown colloids were obtained by washing alternately with deionized water and ethanol five times, and then dried overnight at 60 °C.

### 4.3. Synthesis of C/TiO_2_ Calcined Xerogels

C/TiO_2_ calcined xerogels were prepared via the sol–gel–calcination assisted method by employing MCSs as the template. Briefly, 0.2 g of MCS powders were dispersed into 100 mL of absolute ethanol, and then 4 mL of TBOT was added under vigorous stirring for 2 h. Subsequently, 120 mL of deionized water was added dropwise to the suspension with stirring for an additional 12 h. The resulting gels were centrifuged, washed five times with ethanol, and then oven-dried at 60 °C overnight. In order to construct C/TiO_2_ yolk–shell and TiO_2_ hollow structure, the xerogels were calcined inside a muffle furnace in air at 500 °C for 30 and 50 min, respectively. Meanwhile, the core–shell structured C/TiO_2_ composite was obtained by calcination in an Ar atmosphere at 500 °C for 30 min.

### 4.4. Characterization

The microstructure and morphology of the synthesized photocatalysts were characterized by X-ray diffraction patterns (XRD, SmartLab), Raman spectrometry (Renishaw, inVia Qontor), field emission scanning electron microscopy (FESEM, JSM-6700F model, JEOL, Tokyo, Japan), and transmission electron microscopy (TEM, JEM2100F model, JEOL, Tokyo, Japan) with dual-energy dispersive X-ray spectrometers. Thermal gravimetric analysis (TGA) was performed using a TGA/DSC 1/1600 analyzer (Mettler Toledo, Greifensee, Switzerland) from room temperature to 600 °C at a heating rate of 10 °C min^−1^ under an air atmosphere. Ultraviolet-visible diffuse reflectance spectra (UV–Vis DRS) were recorded on a PerkinElmer Lambda 950 spectrometer (Shelton, CT, USA). PL spectra were studied by an Edinburgh FLS980 fluorescence spectrophotometer (Livingston, UK) at room temperature. Surface photovoltage (SPV) spectra were observed on a self-assembly apparatus [39,40]. The photoelectrochemical measurements of transient photocurrent responses and electrochemical impedance spectroscopy (EIS) were recorded on a three-electrode photoelectrochemical cell of the CHI660E workstation using a 300 W xenon lamp (CHF-XM300W, PerfectLight, Beijing, China) as a light source.

### 4.5. Photocatalysis

Photocatalytic H_2_ production experiments were carried out in a self-designed 25 mL visual high-pressure reactor (Xi’an Taikang Biological Technology Co., Ltd. (Xi’an, China) [39,40] under room temperature maintained via external circulating water. Initially, 10 mg of the synthesized samples was added and dispersed into 5 mL of TEOA (10 vol%) aqueous solution. After sealing, the reactor was deaerated through bubbling pure N_2_ gas (99.99%) thoroughly for 30 min to remove air. Then, the reactor was illuminated by a 300 W Xenon lamp (100 mW/cm^2^, CHF-XM300W, PerfectLight) through the transparent window on the top of the visible reactor. Finally, the evolved H_2_ was sampled and quantified at 1 h intervals using an Agilent 8890 Gas Chromatograph (Wilmington, DE, USA) equipped with a 5 Å molecular sieve column and a thermal conductive detector (TCD) with high purity nitrogen N_2_ (99.999%) flow as the carrier gas.

## Data Availability

The data related to this paper may be requested from the corresponding authors.

## Supplementary Materials

The following supporting information can be downloaded at: https://www.mdpi.com/article/10.3390/gels11110911/s1, Figure S1: TG curves for thermal decomposition of various calcined C/TiO_2_ xerogels; Figure S2: (a) SEM image of YS-C/TiO_2_ after five consecutive photocatalytic cycles; (b) XRD patterns of YS-C/TiO_2_ before and after the five-cycle test; Table S1: Hydrogen evolution of C/TiO_2_-based photocatalysts from water reported in the literature [40,44,45,46,47,48,49,50,51,52,53,54,55,56].
