# Supplementary material for: Calcined Xerogels of C/TiO2 Nanostructures for Solar-Driven Photocatalytic Hydrogen Production
Source: Gels. 2025 Nov 14;11(11):911. doi: 10.3390/gels11110911 (PMC12652275; doi:10.3390/gels11110911)
Supplement: Supplementary file 1 [file gels-11-00911-s001.zip › gels-3949713-supplementary.pdf]

Supplementary Material

Calcined Xerogels of C/TiO_2 Nanostructures for Solar-Driven Photocatalytic Hydrogen Production

Yong Li [1,2,*], Hongpeng Zhang [1], Canni Zhuo [1], Xixi Sun [1], Jiaqi Gao [1,2] and Yali Zhao [1,2,*]

[1] Department of Materials Science and Engineering, Jinzhong University, Jinzhong 030619, China; 15635424073@163.com (H.Z.); zhuocanni111@163.com (C.Z.); k1224122chris@163.com (X.S.); gaojiaqi@jzxy.edu.cn (J.G.)
[2] Shanxi Province New Multi-Functional Glass Technology Innovation Center, Jinzhong University, Jinzhong 030619, China
* Correspondence: liyong@jzxy.edu.cn (Y.L.); zhaoyl@jzxy.edu.cn (Y.Z.)

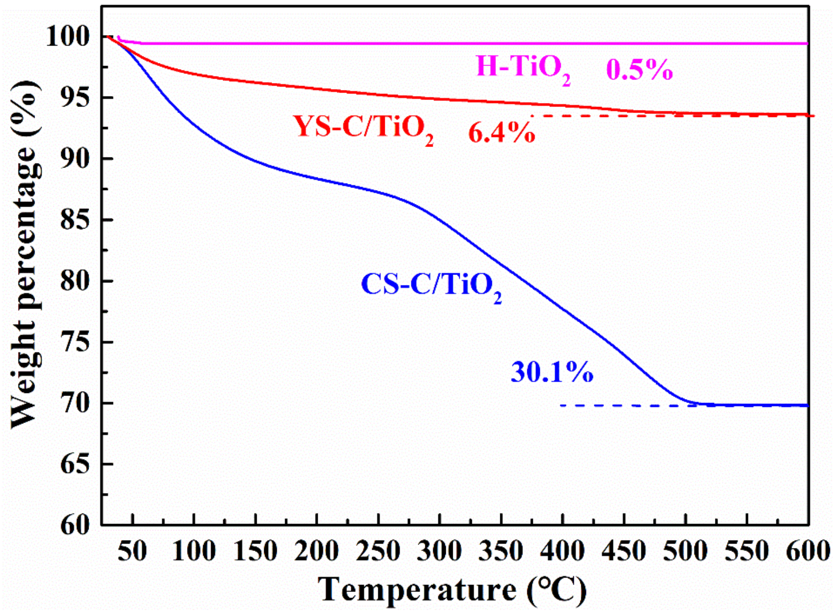

Figure S1. TG curves for thermal decomposition of various calcined C/TiO_2 xerogels

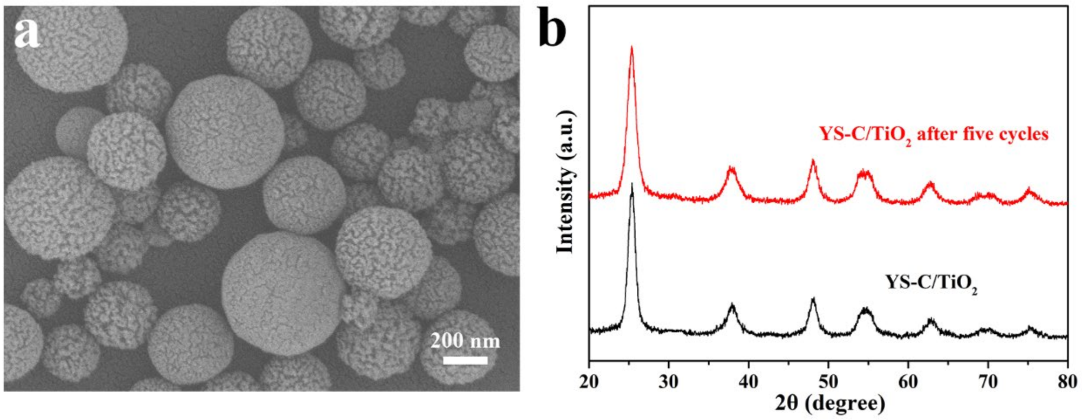

Figure S2. (a) SEM image of YS-C/TiO_2 after five consecutive photocatalytic cycles; (b) XRD patterns of YS-C/TiO_2 before and after the five-cycle test.

Table S1. Hydrogen evolution of C/TiO_2-based photocatalysts from water reported in literatures.

Catalyst	Cocatalyst	Sacrificial agent	Light source	HER (μmol g^{-1} h^{-1})	Ref.
**YS-C/TiO_2**	**N/A**	**TEOA**	**300W Xenon lamp**	**975**	**This work**
CDs/TiO_2	N/A	Ethanol	300 W Xenon lamp	543.6	[44]
Hollow C@TiO_2	N/A	Methanol	300 W Xenon lamp	533	[45]
CQDs/P25	N/A	Methanol		182	[46]
VTi@CQDs@rGO	N/A	Methanol		638	[47]
B-TiO_2/g-C_3N_4	N/A	TEOA	300W Xe arc lamp	808.97	[48]
Cu-TiO_2@C	N/A	Methanol	300 W Xenon lamp	269.1	[49]
Cr_2O_3/C@TiO_2	N/A	Methanol	300 W Xenon lamp	446	[50]
C@TiO_2/TiO_{2-x}	N/A	TEOA	300W Xe lamp AM 1.5G filter	3667	[40]
C@TiO_{2-x}/CNNS	3 wt.% Pt	TEOA	300 W AM 1.5G	1830.93	[51]
g-CS@TiO_{2-x}	1 wt.% Pt	0.5 M Na_2S and 0.5 M Na_2SO_3	UV-LEDs (3 × 4 W, 420 nm)	255.2	[52]
Ru/TiO_2	N/A	N/A	300W Xe lamp AM 1.5G filter	40.2	[53]
CaF_2O_4/TiO_2	N/A	N/A		92	[54]
Co_3O_4/TiO_2	N/A	N/A	300W Xe lamp	41.8	[55]
TiO_2(B)/RP	Pt	N/A		380	[56]
C@TiO_2/TiO_{2-x}	N/A	N/A	300W Xe lamp AM 1.5G filter	336	[40]
**YS-C/TiO_2**	**N/A**	**N/A**	**300W Xenon lamp**	**102**	**This work**
